# Supplementary material for: ARROW: Allele-Specific Recombined gRNA Design for Reduced Off-Target with Enhanced Specificity
Source: Bioengineering (Basel). 2025 Nov 12;12(11):1237. doi: 10.3390/bioengineering12111237 (PMC12649741; doi:10.3390/bioengineering12111237)
Supplement: Supplementary file 1 [file bioengineering-12-01237-s001.zip › bioengineering-3934977-supplementary.pdf]

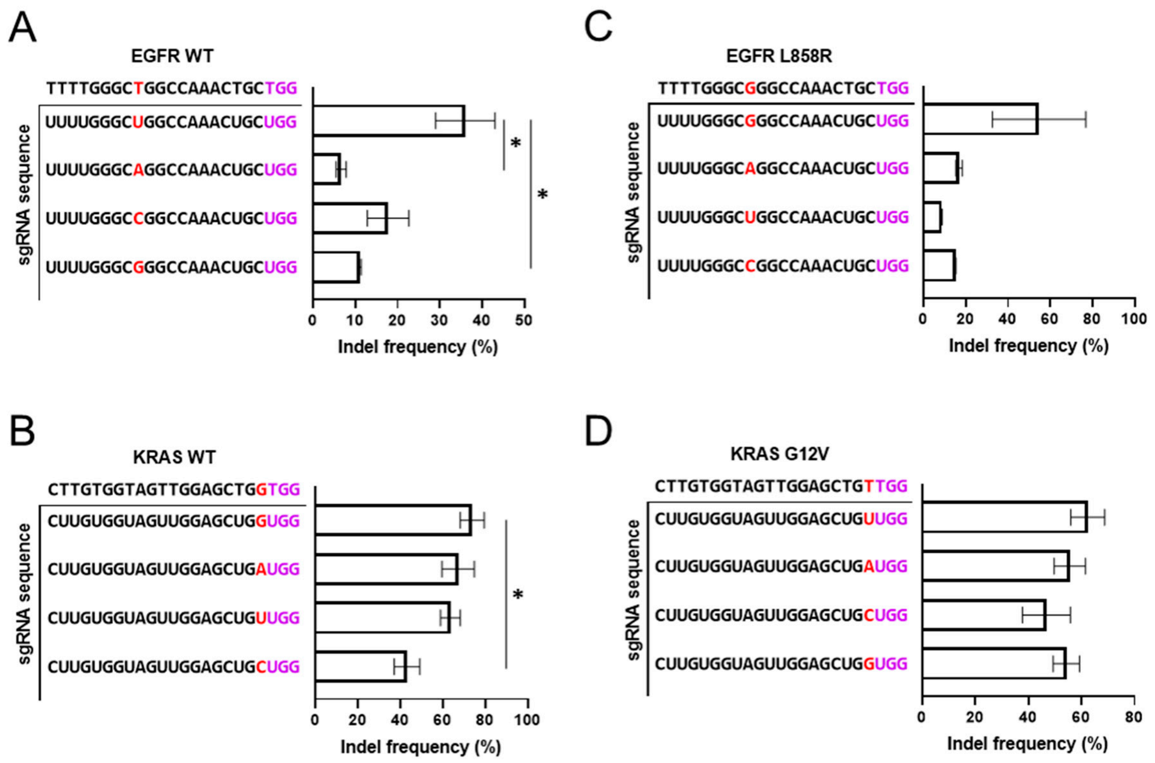

Supplementary Figure S1. Characterization of the indel rates of gRNA variants on EGFR L858R and KRAS G12V mutations

The indel rates of matched and mismatched gRNAs to wild-type or mutant alleles of EGFR and KRAS genes are analyzed via dual fluorescence reporter vector system. In all target genes, despite a single base difference, the gRNAs still cleaved (A, B) wild-type and (C, D) mutant sequences of EGFR and KRAS. Statistical significance is denoted with asterisks (*, P<0.05).

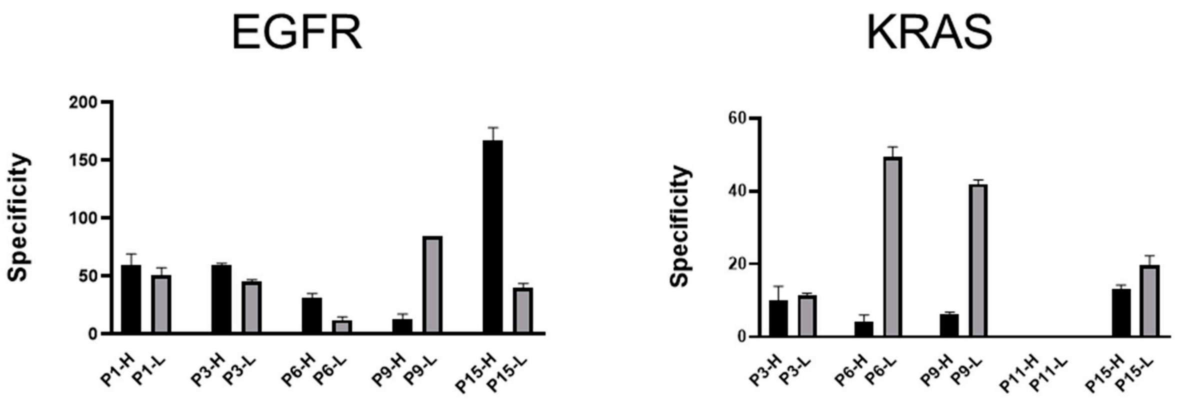

Supplementary Figure S2. Evaluation of specificity of mismatched gRNAs targeting EGFR and KRAS mutations

The graphs compare the specificity of mismatched gRNAs from indel rates data on figure 4. Although the indel rates on the mutant allele was reduced by introducing an additional single-base mismatch, the indel rates on the wild-type allele was decreased even more substantially, there by resulting in enhancement of specificity.
